# Liquid Biopsy Biomarkers in Patients With Metastatic Castration-Resistant Prostate Cancer Receiving Prostate-Specific Membrane Antigen Radioligand Therapy: Protocol for a Prospective, Longitudinal Multicenter Observational Study

**DOI:** 10.2196/86737

**Published:** 2026-04-29

**Authors:** Kerstin Michalski, Theo Lorenzini, Tim Jedamzik, Marieke Heinrich, Aleksander Kosmala, Isabel Rauscher, Mika Bauch, Lena Reichl, Luisa Amann, Simon Liebl, Andreas Buck, Matthias Eiber, Constantin Lapa, Rainer Claus

**Affiliations:** 1 Department of Nuclear Medicine, University Hospital Würzburg, Oberdürrbacher Str. 6, Würzburg, 97080, Germany; 2 Bavarian Cancer Research Center (BZKF), Erlangen, Germany; 3 Department of Nuclear Medicine, School of Medicine and Health, Technical University of Munich, Munich, Germany; 4 Hematology and Oncology, Faculty of Medicine, University Hospital Augsburg, Augsburg, Germany; 5 Department of Nuclear Medicine, Faculty of Medicine, University Hospital Augsburg, Augsburg, Germany; 6 Pathology, Faculty of Medicine, University of Augsburg, Augsburg, Germany

**Keywords:** prostate cancer, prostate-specific membrane antigen radioligand therapy, PSMA radioligand therapy, liquid biopsy, circulating tumor DNA, biomarker, personalized medicine

## Abstract

**Background:**

Prostate cancer is the second leading cause of cancer-related mortality in men worldwide. Prostate-specific membrane antigen (PSMA)–targeted radioligand therapy (RLT) has emerged as a theranostic strategy for metastatic castration-resistant prostate cancer (mCRPC), with [^177^Lu]Lu-PSMA-617 demonstrating survival benefits in the VISION trial. However, clinical responses are heterogeneous, and resistance mechanisms remain poorly understood. Liquid biopsy (LBx), particularly circulating tumor DNA (ctDNA), may provide a minimally invasive approach to assess tumor heterogeneity, monitor response, and detect emerging resistance.

**Objective:**

The LOOPS (Liquid Biopsy as a Biomarker in Patients Treated with PSMA Radioligand Therapy) study aims to prospectively investigate the prognostic and predictive value of ctDNA in patients undergoing PSMA RLT.

**Methods:**

LOOPS is a prospective, multicenter observational biomarker trial recruiting 100 patients with mCRPC eligible for PSMA RLT across 3 Bavarian cancer research centers in Germany. Patients will undergo up to 6 cycles of [^177^Lu]Lu-PSMA-617. Blood samples for ctDNA analysis will be collected longitudinally (at baseline and after cycles 1, 2, 4, and 6) and processed according to standardized protocols. Imaging with PSMA positron emission tomography–computed tomography and clinical and biochemical data will be systematically collected. The primary end points are the prognostic and predictive value of baseline ctDNA for treatment response, defined by imaging- and prostate-specific antigen (PSA)–based response criteria after 2 cycles of PSMA RLT. Predictive performance will be evaluated using receiver operating characteristic analyses, including the determination of optimal ctDNA cutoffs, sensitivity, specificity, positive and negative predictive values, and multivariable logistic regression models adjusting for relevant clinical confounders. Secondary end points assess the concordance and correlation between ctDNA dynamics, systematically assessed PSMA positron emission tomography–computed tomography response, and PSA changes, as well as associations between ctDNA and clinical or laboratory (eg, age, Gleason score, and other) characteristics at baseline and during follow-up. Exploratory analyses will investigate early molecular response patterns based on short-term ctDNA changes and will characterize clonal dynamics and potential resistance mechanisms under therapy.

**Results:**

The study has been funded by the German Research Foundation (Deutsche Forschungsgemeinschaft) since July 2025. Patient recruitment has already commenced, and in January 2026, a total of 63 patients have been enrolled. Analysis of LBx samples was initiated in January 2026, while recruitment and data collection are ongoing.

**Conclusions:**

The LOOPS study will provide the first prospective, systematic evaluation of ctDNA as a biomarker in PSMA RLT. By integrating molecular, imaging, and clinical data, it aims to clarify the role of LBx in response monitoring and the early identification of resistance. The results could pave the way toward personalized therapeutic strategies in mCRPC.

## Introduction

### Background

Prostate cancer is the second leading cause of cancer-related death in men worldwide [1]. Despite therapeutic advances, many patients progress to metastatic castration-resistant prostate cancer (mCRPC), a stage with limited therapeutic options and poor prognosis. Prostate-specific membrane antigen (PSMA) has emerged as a pivotal molecular target for imaging and therapy. PSMA expression is upregulated up to 1000-fold in prostate cancer cells and increases with tumor dedifferentiation, making it particularly relevant in mCRPC [2,3]. This biology enables theranostic strategies integrating molecular imaging and targeted radioligand therapy (RLT). The clinical implementation of PSMA-targeted RLT represents a significant therapeutic advance. [^177^Lu]Lu-PSMA-617, approved by the Food and Drug Administration and European Medicines Agency in 2022, demonstrated survival benefits in the pivotal VISION trial, prolonging both overall survival (15.3 vs 11.3 months; hazard ratio 0.62; *P*<.001) and radiographic progression-free survival (8.7 vs 3.4 months; hazard ratio 0.40; *P*<.001) compared to the standard of care alone [4].

### Unmet Need

Despite these advances, response heterogeneity and the ability to predict response remain major challenges. Only approximately 50% of patients achieved an objective radiographic response in the VISION trial [4]. This discordance between high PSMA expression on positron emission tomography (PET)–computed tomography (CT) and limited efficacy suggests incompletely understood resistance mechanisms, including tumor microenvironment heterogeneity, insufficient radiation doses to micrometastases, and intrinsic molecular factors such as genetic alterations in DNA damage repair pathways [5,6]. Liquid biopsy (LBx) has emerged as a promising tool that enables longitudinal, minimally invasive monitoring while mitigating the sampling bias of single-site tissue biopsies [7]. LBx analytes include circulating tumor cells (CTCs), circulating tumor DNA (ctDNA), extracellular vesicles, and tumor-derived RNA [8]. Among these, ctDNA is particularly promising in mCRPC. First, it is detectable in a substantial majority of patients (approximately 76%‐94%), with lower detection in patients with limited disease burden or low prostate-specific antigen (PSA) levels [9-11]. Second, ctDNA levels strongly correlate with clinical outcomes, including PSA response, progression-free survival, and overall survival, as demonstrated in multiple studies, including the TheraP trial [12,13]. Third, ctDNA provides a comprehensive view of the tumor genomic landscape, enabling detection of actionable mutations at higher rates than CTC-based assays or single-site tissue biopsies [14]. Recent studies have demonstrated specific genomic alterations detectable in ctDNA that predict resistance to PSMA RLT, including amplifications of *FGFR1* and *CCNE1*, as well as *CDK12* mutations [15]. Additionally, genome-wide copy number variation analysis has shown that a higher burden of genomic instability correlates with poorer treatment response [16]. Finally, ctDNA has been shown to correlate with imaging-derived biomarkers such as PSMA-positive tumor volume on PET, with correlations being particularly strong in patients with castration-resistant cancer (*r*=0.65; *P*<.001) compared to hormone-sensitive patients [11]. In contrast, early LBx applications in PSMA RLT using CTCs showed limited predictive value [17] and a 20% false-negative rate for PSMA expression [18].

Knowledge gaps remain regarding optimal ctDNA thresholds, the timing and magnitude of early on-treatment ctDNA dynamics that foretell radiographic progression, genomic correlates of resistance despite high PSMA expression, and the optimal timing for sampling. The LOOPS (Liquid Biopsy as a Biomarker in Patients Treated with PSMA Radioligand Therapy) trial is a multicenter, prospective biomarker trial in patients initiating PSMA RLT, designed to integrate serial ctDNA profiling with PSMA PET-CT, biochemical, and clinical end points to address the unmet need for predictive and prognostic biomarkers. The objectives are to assess whether baseline ctDNA tumor fraction and early ctDNA changes predict radiographic response, quantify the concordance between ctDNA burden and PSMA PET tumor volume, and identify ctDNA-detectable genomic alterations linked to resistance to PSMA RLT. To enhance analytical rigor, we will address clonal hematopoiesis (through paired leukocyte sequencing or bioinformatic filtering), standardize preanalytical handling, and report the assay limit of detection and reproducibility. By establishing fit-for-purpose ctDNA biomarkers, the LOOPS study (registered on August 11, 2025) aims to enable risk-adapted, personalized PSMA RLT in mCRPC. We hypothesize that baseline ctDNA burden and genomic features are prognostic for early nonprogression under [^177^Lu]Lu-PSMA-617 and that on-treatment ctDNA dynamics provide an early, quantitative readout of therapeutic efficacy that aligns with PSMA PET-CT, PSA responses, and clinical outcomes. Exploratory analyses address clonal evolution and resistance mechanisms emerging under the selective pressure of RLT.

## Methods

### Study Design

LOOPS is a prospective, multicenter, observational biomarker study embedded in routine care. No investigational treatment is introduced; rather, standard-of-care [^177^Lu]Lu-PSMA-617 is augmented by serial LBx sampling and centralized imaging reads.

### Study Setting

Three university hospitals within the Bavarian Cancer Research Center—Würzburg, Munich (Technical University of Munich [TUM] Klinikum), and Augsburg—participate. These centers collectively manage several hundred PSMA RLT cases annually, supporting feasibility and external validity.

### Eligibility Criteria

Adults (aged ≥18 years) with histologically confirmed prostate cancer that has progressed to mCRPC are eligible if they are candidates for PSMA RLT within approved indications, have previously received at least 1 androgen-receptor pathway inhibitor and 1 taxane-based chemotherapy, and have PSMA PET-CT within 8 weeks prior to cycle 1 demonstrating adequate target expression. Prior exposure to taxane-based chemotherapy is required as an inclusion criterion in accordance with regulatory approvals and the standard-of-care indication for PSMA-targeted RLT at the time of study design. This criterion ensures that all enrolled patients are treated within the approved clinical framework and reflects real-world treatment sequencing in mCRPC. Patients must provide written informed consent and must either be German speaking or have a translator available. Exclusion criteria are limited to the inability or unwillingness to adhere to study procedures and the absence of an interdisciplinary tumor board recommendation for PSMA RLT.

### Interventions

Therapy follows routine clinical practice: [^177^Lu]Lu-PSMA-617 is administered intravenously every 6 weeks for up to 6 cycles, with initiation, delay, or discontinuation at the discretion of the treating physician. The study does not modify dosing or scheduling.

### Outcomes

Two coprimary outcomes are specified. The first is the prognostic value of baseline ctDNA for nonprogression after 2 cycles, as assessed by PSA response according to PCWG3 (Prostate Cancer Clinical Trials Working Group 3) criteria [19]) and by RECIP 1.0 (Response Evaluation Criteria in PSMA-imaging, version 1.0) [20,21] defined as partial response or stable disease vs progression. The second is the concordance between ctDNA dynamics (percent change from baseline) and imaging-based response according to RECIP 1.0, as well as PSA response, after cycles 2, 4, and 6. Secondary outcomes examine associations between baseline ctDNA features and clinical or laboratory parameters (eg, age, Gleason score, PSA, alkaline phosphatase, lactate dehydrogenase, and metastatic distribution) and relate longitudinal ctDNA changes to clinical and laboratory trajectories. Exploratory outcomes include a very early molecular response defined a priori as a ≥30% decline in ctDNA at 6 weeks after the first cycle and the characterization of clonal dynamics and resistance-associated alterations across multiple time points.

### Participant Timeline

**Figure 1.**
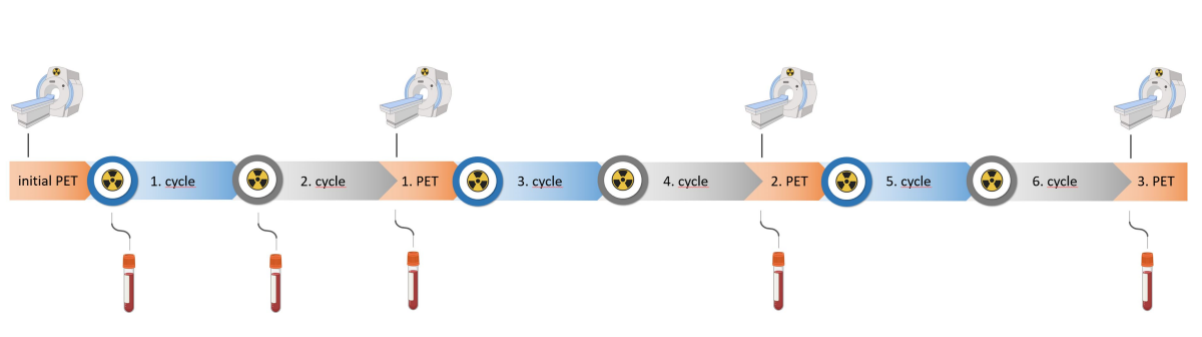
Time points of blood sampling for liquid biopsy in the LOOPS (Liquid Biopsy as a Biomarker in Patients Treated with Prostate-Specific Membrane Antigen Radioligand Therapy) study. PET: positron emission tomography.

Participants are enrolled before cycle 1. Blood sampling for ctDNA occurs at baseline (≤14 days prior to cycle 1); before cycle 2 (approximately 6 weeks); and at imaging visits after cycle 2 (approximately 16 weeks), cycle 4 (approximately 28 weeks), and cycle 6 (approximately 40 weeks). An exploratory time point at 6 weeks after the first cycle enables the assessment of very early molecular response. PSMA PET-CT is obtained at baseline and after cycles 2, 4, and 6 and submitted for central review (Figure 1). All patients are treated with the approved PSMA RLT according to the manufacturer’s recommendations; accordingly, the treatment schedule and administered activity are fixed.

### Sample Size

The planned sample size of 100 patients is based on a priori biometrical considerations and reflects expected feasibility in this population with advanced mCRPC. Due to disease severity and anticipated attrition, the primary end point (treatment response at first posttherapy restaging after 2 cycles of therapy) is expected to be evaluable in approximately 70 patients. On the basis of this effective sample size, power calculations demonstrate that clinically meaningful differences in response rates between patients with and without a ctDNA-based predictive marker or marker change can be detected with 80% power at a 2-sided significance level of α=.05. Specifically, depending on the prevalence of the marker or marker change (25%, 50%, or 75%), differences in response probabilities of 0.33 to 0.41 between marker-positive and marker-negative patients are detectable using Fisher exact test. These effect sizes correspond to sensitivities ranging from 38% to 88% and specificities from 44% to 93%, supporting the ability of the study to evaluate the prognostic and predictive performance of ctDNA metrics. Therefore, the planned sample size is considered adequate for the primary analyses while remaining consistent with the pragmatic, multicenter, observational design of the study. To control the family-wise error rate for the 2 coprimary end points, a hierarchical testing strategy will be applied. The association between baseline ctDNA and treatment response will first be tested at a 2-sided significance level of α=.05. Only if this test is statistically significant, the discriminative performance (receiver operating characteristic analysis and area under the curve) will be formally evaluated at the same α level.

### Recruitment

Eligible patients are identified during routine evaluation for PSMA RLT at participating centers. Study information is provided during treatment planning, and informed consent is obtained prior to baseline sampling.

### Data Collection Methods

#### Biospecimens and Preanalytical Procedures

Peripheral blood is collected in ethylenediaminetetraacetic acid tubes (5×9 mL at baseline and 3×9 mL at follow-up visits), processed within 2 hours, double-spun, aliquoted, and stored decentrally at −80 °C. Preanalytical procedures are harmonized across sites using standard operating procedures adapted from the Augsburg Longitudinal Plasma Study [17], with a barcode-based chain-of-custody. Matched leukocyte DNA is collected to facilitate the filtering of germline variants and clonal hematopoiesis.

#### ctDNA Workflow

Centralized analyses are performed at the Interdisciplinary Laboratory for Experimental Cancer Research, Augsburg. Cell-free DNA is extracted from approximately 4 mL of plasma and quantified with assessment of fragment size distribution. Libraries are prepared using the AVENIO ctDNA Surveillance V2 panel (197 genes, including National Comprehensive Cancer Network–listed prostate cancer genes with unique molecular identifiers for error suppression) and sequenced using paired-end 100 base pairs reads on an Illumina NovaSeq X to >20,000×raw coverage. Bioinformatics identifies single nucleotide variants or indels and copy number alterations; ctDNA burden is summarized as variant allele fractions and human genome equivalents. ΔctDNA is defined as the percent change from baseline at each on-treatment time point. Variants consistent with germline origin and clonal hematopoiesis (eg, *DNMT3A*, *TET2*, and *ASXL1*) are annotated and excluded from tumor-specific analyses. Clonal hematopoiesis of indeterminate potential (CHIP) refers to the age-related expansion of hematopoietic cell clones carrying recurrent somatic mutations (often in genes such as *DNMT3A*, *TET2*, and *ASXL1*) [1]. DNA from these CHIP-derived clones can be released into the bloodstream and detected in plasma, where such mutations may be mistakenly attributed to the tumor, potentially confounding ctDNA analysis. Therefore, filtering out CHIP-associated variants via matched leukocyte DNA sequencing is essential to avoid false-positive results and ensure that ctDNA findings truly reflect tumor-derived alterations. Prespecified quality metrics and reporting thresholds are applied uniformly.

#### Imaging

PSMA PET-CT is performed according to institutional SOPs with locally available radiotracers. PET-CT imaging is performed at 3 centers using different PET-CT systems and protocols. In Wurzburg, scans are acquired on a Siemens Biograph mCT 64 or a Siemens Biograph mCT 128 Flow scanner. At 90 minutes after the injection of [¹⁸F]PSMA-1007 (3 MBq/kg body weight), patients undergo a whole-body PET acquisition with a scan duration of 2 minutes per bed position. In Augsburg, PET-CT scans are performed on a Siemens Biograph mCT 40 or a GE Discovery Molecular Imaging system. Patients receive approximately 4 MBq/kg body weight of [¹⁸F]rhPSMA-7.3 or [¹⁸F]DCFPyL (Pylarify), and imaging is conducted 60 minutes after injection with an acquisition time of 2 minutes per bed position. In Munich, PET scans are acquired on 2 hybrid PET-CT systems (Biograph molecular CT and Biograph Vision 600; Siemens). The radiotracer is predominantly [¹⁸F]rhPSMA-7.3, with [¹⁸F]PSMA-1007 used alternatively during cyclotron maintenance, and the injected activity is 3 MBq/kg body weight. Imaging is performed 60 minutes after injection for [¹⁸F]rhPSMA-7.3 and 90 minutes after injection for [¹⁸F]PSMA-1007, with an acquisition time of 3 to 4 minutes per bed position or a flow acquisition speed of 1.1 to 1.5 mm/s. In follow-up examinations, the administered radiotracer may differ from that used at baseline, depending on tracer availability and local clinical protocols. All scans are pseudonymized and transferred for central evaluation at Wurzburg. Response will be assessed by visual RECIP 1.0 relative to baseline after cycles 2, 4, and 6 by 1 reader. In case of a change of radiotracer during therapy, 2 readers in consensus will perform a visual assessment to minimize the potential effects of differences in visual PSMA-positive tumor volume, and the number of patients with a change in radiotracers will be stated in the final manuscript.

#### Clinical Data

Demographics, baseline tumor characteristics (Gleason score and metastatic sites), laboratory values (eg, PSA, lactate dehydrogenase, and alkaline phosphatase), and treatment details (ie, cycles administered and administered activity) are captured prospectively and pseudonymized before transfer to the central database for integration with ctDNA and imaging data.

### Data Management

All original source documents and records required for patient care during PSMA RLT are retained in the patients’ medical files at the respective treating hospitals. In accordance with the Radiation Protection Ordinance, these records are stored for a minimum of 30 years. LBx results are archived for 30 years at the Interdisciplinary Laboratory for Experimental Cancer Research. Clinical and laboratory data from the participating nuclear medicine departments (Wurzburg, Augsburg, and TUM Munich) are centrally combined in an electronic data capture master file at the University Hospital Wurzburg and made available to statistical collaboration partners for analysis. Pseudonymized PSMA PET-CT data are transferred in Digital Imaging and Communications in Medicine format to the Department of Nuclear Medicine, University Hospital Wurzburg, and stored together with the clinical and laboratory data for a period of 10 years. Written informed consent, including approval for pseudonymized data transfer among the collaborating centers, is obtained at each participating site and stored in the respective local study documentation. All study results are published in anonymized form in peer-reviewed journals, and anonymized research data may be made available to third parties upon reasonable request.

### Statistical Methods

The primary analyses evaluate the prognostic and predictive value of baseline ctDNA for treatment response after 2 cycles of PSMA RLT, as defined by imaging- and PSA-based criteria. Predictive performance is assessed using receiver operating characteristic analyses, including the determination of optimal ctDNA cutoffs, as well as sensitivity, specificity, positive and negative predictive values, and multivariable logistic regression models adjusting for relevant confounders.

Treatment response is further assessed by analyzing longitudinal changes in ctDNA levels and their concordance with PSMA PET-CT and PSA responses, using the Cohen κ coefficient and correlation analyses. Secondary analyses investigate associations between baseline and longitudinal ctDNA metrics and clinical or laboratory parameters, including subgroup comparisons based on patient characteristics. Exploratory analyses focus on the identification of very early molecular responders based on short-term ctDNA changes and on the characterization of clonal evolution and potential resistance mechanisms using deep sequencing and computational clonal deconvolution methods. General statistical considerations include the handling of missing data, adjustment for multiple testing, and sensitivity analyses to ensure robustness of the results. Given that tumor burden and ctDNA levels may change over relatively short time intervals in heavily pretreated mCRPC, the temporal separation between baseline PSMA PET-CT imaging and baseline blood sampling will be accounted for in the statistical analysis. The exact interval (in days) between PET-CT and blood draw will be recorded for all patients. The primary analysis correlating baseline ctDNA with PET-derived tumor burden will be prespecified to include only patients with a PET-to-blood interval of ≤28 days. In addition, sensitivity analyses will be performed in the full cohort, incorporating the PET-to-blood interval as a covariate (modeled as a continuous variable and as a categorical variable ≤28 vs 29‐56 days). Patients with intervals exceeding 28 days will be described separately, and the impact of their inclusion vs exclusion on key estimates will be explicitly reported. Furthermore, we will assess the association between cumulative administered activity and longitudinal changes of ctDNA. Cumulative activity will be modeled as a continuous exposure variable and analyzed in relation to ctDNA dynamics using regression-based approaches. Landmark analyses will be used where appropriate to mitigate immortal time bias.

### Oversight and Monitoring

Oversight is provided by local principal investigators at each site and a central coordinating team. As an observational study embedded in routine care, no independent data monitoring committee is planned; safety events related to standard-of-care therapy are recorded per institutional policy.

### Harms

No study-specific interventions are introduced. Adverse events associated with routine [^177^Lu]Lu-PSMA-617 administration are documented and summarized descriptively.

### Ethical Considerations

The study received ethics approval from the committees of Wurzburg (180/23), TUM Munich (2024-415-S-CB), and Augsburg–Ludwig-Maximilians-Universität Munich (24-0707). All participants provide written informed consent prior to enrollment. The study is conducted in accordance with the Declaration of Helsinki and Good Clinical Practice. Participation in the study is voluntary and without financial compensation. All clinical data, images, and biospecimens are pseudonymized at source with role-based access and audit logs. Biospecimens are barcoded and stored under controlled conditions; shipments follow applicable regulations. This study was registered on ClinicalTrials.gov (NCT07118436) on August 11, 2025. Registration details conform to the World Health Organization Trial Registration Data Set.

### Dissemination Policy

Results will be disseminated through peer-reviewed publications and conference presentations. Summary findings will be shared with participating centers; deidentified datasets and analysis code may be made available upon reasonable request and in line with regulatory and institutional policies.

## Results

The study has been funded by the German Research Foundation (Deutsche Forschungsgemeinschaft) since July 2025. Patient recruitment has commenced and is ongoing; as of January 2026, a total of 63 patients have been enrolled. Completion of recruitment is planned for September 2026, with a target sample size of 100 patients. This timeline is designed to ensure that all enrolled patients will have completed restaging after the second cycle of PSMA RLT by the end of 2026, enabling evaluation of the primary study end point. Analysis of LBx samples was initiated in January 2026 and will continue in parallel with recruitment and follow-up. Publication of the primary end point is planned for mid-2027.

## Discussion

### Anticipated Findings

LOOPS is, to our knowledge, the first prospective, multicenter, observational study to systematically evaluate ctDNA alongside PSMA PET-CT and clinical or laboratory measures in men with mCRPC receiving PSMA-directed RLT. Against the background of the proven benefit of [^177^Lu]Lu-PSMA-617 in the VISION trial [4], the persistence of heterogeneous responses highlights the need for biomarkers that stratify prognosis and monitor on-treatment effectiveness early in the course of therapy.

PSMA PET-CT has become essential for patient selection in RLT; however, it cannot fully capture prostate cancer’s biologic complexity. Although imaging parameters such as maximum standardized uptake value and total tumor volume provide valuable information, they cannot reflect underlying genomic alterations that may drive therapy resistance [5,6]. ctDNA, in contrast, samples tumor-derived alterations across metastatic sites and has been reported to be detectable in a substantial proportion of patients with mCRPC, with associations with clinical outcomes across studies [9,14]. By prospectively aligning serial ctDNA measurements with RECIP 1.0–defined PET-CT response, LOOPS will quantify the degree to which molecular dynamics and radiographic change provide complementary information. Prior work suggests that PSMA imaging and LBx offer independent, nonredundant prognostic signals [11]. LOOPS is designed to test this synergy using standardized preanalytical procedures, central PET-CT reading, and prespecified end points.

Previously, LBx efforts in this setting focused on CTCs and were limited by frequent PSMA-negative CTCs despite PSMA-avid disease on imaging [17,18]. Targeted ctDNA profiling has, in contrast, identified genomic features associated with nonresponse (eg, select copy number gains and DNA damage response alterations) and broader copy number instability signals linked to poorer outcomes [9,10,15,16].

LOOPS extends this literature by serially sampling ctDNA to characterize early molecular change after treatment initiation and evolutionary dynamics under therapeutic pressure, while correlating these signals with quantitative PET metrics (eg, total PSMA-positive tumor volume) [5,6,15,16]. These analyses are intended to be hypothesis generating and to inform subsequent interventional studies. By sequencing ctDNA at multiple time points, LOOPS has the potential to identify recurrent mutational patterns associated with treatment failure, potentially paving the way toward combination therapies that overcome resistance.

### Limitations and Challenges

Several methodological challenges must be acknowledged. First, ctDNA detection is not uniform across all patients. Low-shedding tumors or those with limited tumor burden may yield false-negative results. Comparative studies have shown that ctDNA detection rates are significantly lower than PSMA PET detection rates, particularly in hormone-sensitive disease and at low PSA levels [11]. Although LOOPS uses sensitive sequencing and harmonized SOPs, residual variability in detection and quantification is unavoidable.

Second, ctDNA is a systemic signal and cannot localize alterations to specific lesions, potentially obscuring intrapatient heterogeneity evident on PET-CT. Consequently, lesion-restricted resistance mechanisms can be systemically diluted below detection thresholds. This underscores the value of integrating molecular and imaging biomarkers rather than privileging either alone.

Third, attrition is expected in an advanced disease cohort; although the analysis plan anticipates approximately 70 evaluable patients for the primary end point, small numbers in exploratory subgroups will limit power and mandate cautious interpretation with appropriate multiplicity control. However, experience from smaller pilot studies suggests that meaningful associations can be detected even in cohorts of 17 to 44 patients [15,16], providing confidence in the planned sample size.

Fourth, generalizability may be constrained by recruitment within 3 Bavarian academic centers and by variation in locally available PSMA-directed radiotracers; central reading and SOP harmonization mitigate, but do not eliminate, site-level heterogeneity.

Fifth, as an observational study embedded in routine care, LOOPS cannot establish causal effects or test treatment adaptation; early-change analyses will use landmarking to reduce immortal time bias; however, residual confounding may persist.

Sixth, posttherapeutic single-photon emission CT–based dosimetry is not included in the analysis. Although absorbed radiation dose is a biologically relevant determinant of treatment response and resistance, homogeneous dosimetric data are not available due to heterogeneous clinical imaging protocols across the participating centers and the current lack of standardized dosimetry methodologies.

Finally, despite paired leukocyte filtering, clonal hematopoiesis and preanalytical factors remain potential sources of noise.

### Expected Impact and Outlook

Within these constraints, LOOPS is positioned to generate a prospective, multicenter dataset linking serial ctDNA, PSMA PET-CT, and clinical measures in patients with prostate cancer receiving PSMA RLT. If baseline ctDNA burden and early ΔctDNA show consistent associations with imaging and clinical outcomes, these markers could inform risk stratification and offer a practical early readout of treatment effectiveness in routine care. Any move toward clinical implementation would require external validation and, ultimately, interventional studies.

The study leverages the established Bavarian Center for Cancer Research infrastructure and feasibility experience from the Augsburg Longitudinal Plasma Study [22], supporting methodological rigor and translational potential. The resulting dataset should enable predictive modeling and hypothesis generation (eg, candidate thresholds for “molecular response” and genomic correlates of nonresponse) to prioritize for subsequent testing.

Beyond immediate prognostic and monitoring applications, comparing ctDNA dynamics with established PSMA PET-CT biomarkers may help clarify when and how LBx adds value in nuclear medicine workflows for mCRPC. Positive signals from LOOPS would provide the empirical basis for future interventional trials of response-adapted or combination strategies guided by early molecular change, with the goal of improving outcomes while minimizing unnecessary toxicity in likely nonresponders.

Finally, the multicenter design and comprehensive data collection will create a resource for collaborative research, facilitating methodological advances in LBx and supporting broader efforts to refine precision oncology in advanced prostate cancer.

### Conclusions

LOOPS addresses a key unmet need in mCRPC by prospectively linking molecular, imaging, and clinical measures during PSMA RLT. With standardized preanalytical procedures, central PET reads, and prespecified end points, the study is positioned to deliver evidence on the prognostic and monitoring utility of ctDNA and to lay the groundwork for subsequent trials of biomarker-guided PSMA RLT.

## Abbreviations

CHIP: clonal hematopoiesis of indeterminate potential
CT: computed tomography
CTC: circulating tumor cell
ctDNA: circulating tumor DNA
LBx: liquid biopsy
LOOPS: Liquid Biopsy as a Biomarker in Patients Treated with Prostate-Specific Membrane Antigen Radioligand Therapy
mCRPC: metastatic castration-resistant prostate cancer
PET: positron emission tomography
PSA: prostate-specific antigen
PSMA: prostate-specific membrane antigen
RECIP 1.0: Response Evaluation Criteria in Prostate-Specific Membrane Antigen-imaging
RLT: radioligand therapy
TUM: Technical University of Munich

## References

[1] Jemal A, Bray F, Center MM, Ferlay J, Ward E, Forman D (2011). Global cancer statistics. CA Cancer J Clin.

[2] Silver DA, Pellicer I, Fair WR, Heston WD, Cordon-Cardo C (1997). Prostate-specific membrane antigen expression in normal and malignant human tissues. Clin Cancer Res.

[3] O’Keefe DS, Bacich DJ, Heston WD (2004). Comparative analysis of prostate-specific membrane antigen (PSMA) versus a prostate-specific membrane antigen-like gene. Prostate.

[4] Sartor O, de Bono J, Chi KN (2021). Lutetium-177-PSMA-617 for metastatic castration-resistant prostate cancer. N Engl J Med.

[5] Kostos L, Buteau JP, Hofman MS, Azad AA (2023). Determinants of outcome following PSMA-based radioligand therapy and mechanisms of resistance in patients with metastatic castration-resistant prostate cancer. Ther Adv Med Oncol.

[6] Stuparu AD, Capri JR, Meyer CA (2021). Mechanisms of resistance to prostate-specific membrane antigen-targeted radioligand therapy in a mouse model of prostate cancer. J Nucl Med.

[7] Mögele T, Hildebrand K, Sultan A (2025). Dissecting tumor heterogeneity by liquid biopsy-a comparative analysis of post-mortem tissue and pre-mortem liquid biopsies in solid neoplasias. Int J Mol Sci.

[8] Crocetto F, Russo G, Di Zazzo E (2022). Liquid biopsy in prostate cancer management-current challenges and future perspectives. Cancers (Basel).

[9] Wyatt AW, Annala M, Aggarwal R (2017). Concordance of circulating tumor DNA and matched metastatic tissue biopsy in prostate cancer. J Natl Cancer Inst.

[10] Taavitsainen S, Annala M, Ledet E (2019). Evaluation of commercial circulating tumor DNA test in metastatic prostate cancer. JCO Precis Oncol.

[11] Kluge K, Einspieler H, Haberl D (2024). Comparison of discovery rates and prognostic utility of [^68^Ga]Ga-PSMA-11 PET/CT and circulating tumor DNA in prostate cancer-a cross-sectional study. Eur J Nucl Med Mol Imaging.

[12] Hofman MS, Emmett L, Sandhu S (2021). [^177^Lu]Lu-PSMA-617 versus cabazitaxel in patients with metastatic castration-resistant prostate cancer (TheraP): a randomised, open-label, phase 2 trial. Lancet.

[13] Kwan EM, Ng SWS, Tolmeijer SH (2025). Lutetium-177-PSMA-617 or cabazitaxel in metastatic prostate cancer: circulating tumor DNA analysis of the randomized phase 2 TheraP trial. Nat Med.

[14] Tukachinsky H, Madison RW, Chung JH (2021). Genomic analysis of circulating tumor DNA in 3,334 patients with advanced prostate cancer identifies targetable BRCA alterations and AR resistance mechanisms. Clin Cancer Res.

[15] Sartor O, Ledet E, Huang M (2023). Prediction of resistance to ^177^Lu-PSMA therapy by assessment of baseline circulating tumor DNA biomarkers. J Nucl Med.

[16] Kluge K, Haberl D, Haug A (2025). Genomic instability is associated with response to [¹⁷⁷Lu]Lu-PSMA-I&amp;T radioligand therapy: an exploratory, preliminary liquid biopsy analysis. Eur J Nucl Med Mol Imaging.

[17] Kessel K, Seifert R, Weckesser M (2020). Molecular analysis of circulating tumor cells of metastatic castration-resistant prostate cancer patients receiving ^177^Lu-PSMA-617 radioligand therapy. Theranostics.

[18] Derlin T, Riethdorf S, Schumacher U (2023). PSMA-heterogeneity in metastatic castration-resistant prostate cancer: circulating tumor cells, metastatic tumor burden, and response to targeted radioligand therapy. Prostate.

[19] Scher HI, Morris MJ, Stadler WM (2016). Trial design and objectives for castration-resistant prostate cancer: updated recommendations from the Prostate Cancer Clinical Trials Working Group 3. J Clin Oncol.

[20] Gafita A, Rauscher I, Weber M (2022). Novel framework for treatment response evaluation using PSMA PET/CT in patients with metastatic castration-resistant prostate cancer (RECIP 1.0): an international multicenter study. J Nucl Med.

[21] Gafita A, Djaileb L, Rauscher I (2023). Response evaluation criteria in PSMA PET/CT (RECIP 1.0) in metastatic castration-resistant prostate cancer. Radiology.

[22] Sommer S, Schmutz M, Hildebrand K (2024). Concept and feasibility of the Augsburg Longitudinal Plasma Study (ALPS) – a prospective trial for comprehensive liquid biopsy-based longitudinal monitoring of solid cancer patients. J Lab Med.

